# Depression, post-traumatic stress, anxiety, and fear of COVID-19 in the general population and health-care workers: prevalence, relationship, and explicative model in Peru

**DOI:** 10.1186/s12888-021-03456-z

**Published:** 2021-09-17

**Authors:** David Villarreal-Zegarra, Anthony Copez-Lonzoy, Ana L. Vilela-Estrada, Jeff Huarcaya-Victoria

**Affiliations:** 1 Escuela de Medicina, Universidad César Vallejo, Trujillo, Peru; 2 Instituto Peruano de Orientación Psicológica, Lima, Peru; 3 Unidad de Investigación en Bibliometría, Universidad San Ignacio de Loyola, Lima, Peru; 4 Asociación Peruana de Profesionales de las Adicciones, Lima, Peru; 5 CRONICAS Center of Excellence in Chronic Diseases, Universidad Peruana Cayetano Heredia, Lima, Peru; 6 Departamento de Psiquiatría, Hospital Nacional Guillermo Almenara Irigoyen, Lima, Peru; 7 Departamento Académico de Psiquiatría, Universidad Nacional Mayor de San Marcos, Lima, Peru

**Keywords:** Depression, Post-traumatic stress, Anxiety, Fear of COVID-19, Peru

## Abstract

**Background:**

This study has two aims. First, determine the fit of the fear model to COVID-19, anxiety, and post-traumatic stress in the general population and health-care workers. Second, determine which model best explains the relationship between depression and the triad of fear, anxiety, and post-traumatic stress in both groups.

**Method:**

A cross-sectional study was conducted using self-reported questionnaires for anxiety, fear of COVID-19, depression, and post-traumatic stress. Information was collected from adults living in Lima, the capital and the most populous city in Peru. The explanatory models were evaluated using a structural equation model.

**Results:**

A total of 830 participants were included, including general population (*n* = 640) and health-care workers (*n* = 190). A high overall prevalence of depressive symptoms (16%), anxiety (11.7%), and post-traumatic stress (14.9%) were identified. A higher prevalence of depressive, anxious, or stress symptoms was identified in the general population (28.6%) compared to health-care workers (17.9%). The triad model of fear of COVID-19, anxiety, and stress presented adequate goodness-of-fit indices for both groups. A model was identified that manages to explain depressive symptoms in more than 70% of the general population and health-care workers, based on the variables of the triad (CFI = 0.94; TLI = 0.94; RMSEA = 0.06; SRMR = 0.06). In the general population post-traumatic stress mediated the relationship between anxiety and depression (β = 0.12; 95%CI = 0.06 to 0.18) which was significant, but the indirect effect of post-traumatic stress was not significant in health care workers (β = 0.03; 95%CI = − 0.11 to 0.19).

**Limitations:**

The prevalence estimates relied on self-reported information. Other variables of interest, such as intolerance to uncertainty or income level, could not be evaluated.

**Conclusions:**

Our study proposes and tests one model that explains more than 70% of depressive symptoms. This explanatory model can be used in health contexts and populations to determine how emotional factors can affect depressive symptoms.

## Background

Peru is one of the countries most affected worldwide by the COVID-19 pandemic. According to the official data given by the Peruvian Government, up to June 06, 2021, there have been 1,983,570 confirmed cases and 186,511 deaths, and the mortality rate was 9.40% [1]. To ensure a decrease in the spread of the disease, policies, such as isolation and quarantine, have been taken to limit contact and exposure [2]. For instance, on March 16, 2020, Peruvian authorities decreed a state of emergency with mandatory quarantine measures.

The COVID-19 pandemic and the quarantine measures have generated different social and economic problems, which, added to the fear of catching the virus, have affected the mental health of the general population. The prevalence of stress, anxiety, and depression among the general population during the COVID-19 pandemic is estimated to be 29.6, 31.9, and 33.7%, respectively [3]. These figures represent an increase in the prevalence of these mental health disorders compared to pre-pandemic measurements in the general population [3]. Evidence suggests that individuals who have been isolated and quarantined due to COVID-19 have experienced significant levels of anxiety, anger, confusion, and stress [4]. Also, fear of COVID-19 is associated with the presence of anxious depressive symptoms and post-traumatic stress [5].

Fear is one of the most influential factors in the presence of emotional problems, such as anxiety and stress. Evidence from animal models of fear and human studies indicates that exposure to constant fear increases anxiety, which in turn can trigger traumatic stress [6–9]. One possible explanation is that the endocannabinoid system links the perception of external and internal stimuli with different neurophysiological and behavioral outcomes, such as the reaction to fear, anxiety, and stress. This neurobiological mechanism allows the subject to adapt or not to this stress. A traumatic event or a highly stressful situation (i.e. a pandemic) could trigger the emergence of traumatic stress if the fear and anxiety response is not adaptive [7]. Therefore, this relationship can be understood as a sequential process of fear, anxiety, and post-traumatic stress, considering that fear also directly influences the appearance of stress.

### Hypothesis a: triad of fear, anxiety, and post-traumatic stress (see Fig. 1a)

This triad involves short-term or medium-term emotional responses such as the fear and anxiety response to a highly stressful situation (i.e., a pandemic). Fear is defined as the fear or aversion response to a concrete, known or defined situation (i.e., the possibility of catching the virus) [10]. In contrast, the anxiety response is a non-specific, diffuse sensation that is not necessarily associated with concrete or well-defined element (i.e., the context of the pandemic) [10]. Fear, anxiety, and a highly stressful event are elements that correspond to the fear circuit that could trigger acute stress and subsequent post-traumatic stress [10]. Acute stress usually begins immediately after the highly stressful event and lasts from 3 days to 1 month; however, post-traumatic stress can manifest as early as the first month after the event. Therefore, both persons directly exposed to the virus (i.e., healthcare workers) and persons exposed but not exposed but anxious about the uncertainty of the future, the possible infection of family members, and the avalanche of news about COVID-19 (i.e., the general population and healthcare workers) [11], could trigger a state of post-traumatic stress disorder resulting from a highly stressful event such as facing the first few months of the pandemic.

**Fig. 1:**
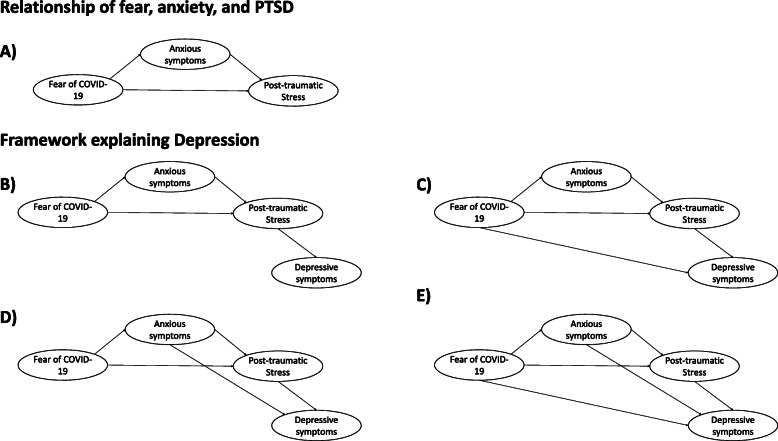
Models that explain the relationship between depression, fear, anxiety, and post-traumatic stress. Note: Figure 1**a** Hypothesis A: Triad of fear, anxiety, and post-traumatic stress. Figure 1**b** Hypothesis B: Post-traumatic stress influences depression. Figure 1**c** Hypothesis C: Fear of COVID-19 influences depression, while anxiety and post-traumatic stress are mediators. Figure 1**d** Hypothesis D: Anxiety influences depression and post-traumatic stress is a mediator. Figure 1**e** Hypothesis E: Fear, anxiety, and post-traumatic stress are related to depression

Although there is ample evidence about the triad of fear, anxiety, and post-traumatic stress, it is not understood how this triad influences the presence of depressive symptoms. Fear of COVID-19 is weakly related to depressive symptoms but more strongly related to anxiety and post-traumatic stress [5]. However, there are several possibilities as to how depression can be explained by the triad of fear, anxiety, and post-traumatic stress (see Fig. 1b-e). Therefore, this study raises four possible hypotheses (hypotheses B, C, D, and E) that could explain the role of depression within this relationship.

### Hypothesis B: post-traumatic stress influences depression (see Fig. 1b)

In many cases, post-traumatic stress is not the only condition resulting from traumatic experiences (i.e., living in a pandemic), but also the onset of other comorbid conditions, including depression, somatization, or physical problems [12]. Longitudinal studies have identified that post-traumatic stress predicts depression [13, 14], so the hypothesis that post-traumatic stress influences depression is plausible, considering that this stress is preceded by the fear of COVID-19 and by anxious symptoms [5].

### Hypothesis C: fear of COVID-19 influences depression, while anxiety and post-traumatic stress are mediators (see Fig. 1c)

This model assumes that the anxious and depressive symptoms are not directly related, but together with post-traumatic stress, they are mediators. However, there is abundant evidence that anxiety and depression are closely related [15, 16]. It is feasible that the force of the relation between anxiety and depression diminishes if post-traumatic stress acts like a mediator [17]. This could support the hypothesis, but it is necessary to prove it with data.

### Hypothesis D: anxiety influences depression and post-traumatic stress is a mediator (see Fig. 1d)

In hypothesis B, the relationship between post-traumatic stress and depression was justified, but in this model, as evidence indicates, post-traumatic stress is considered to be a mediating factor in the relationship between anxiety and depression [18]. Some studies have also identified that post-traumatic stress can mediate depression with other mental health problems. Studies in refugees exposed to different forms of trauma indicate the mediating effect of post-traumatic stress on some mental disorders, such as depression, substance abuse, and personality disorders [19]. In addition, post-traumatic stress can also act as a partial mediator of the relationships between trauma and the severity of depression and between trauma and general mental functioning [20]. Likewise, there is abundant evidence that anxious and depressive symptoms are strongly correlated with each other [15], even in the context of COVID-19 [16]. Therefore, it is suggested that this may be a tentative model of the relationship between depression with fear, anxiety, and post-traumatic stress. For our study, we considered the mediating role of post-traumatic stress since the evidence suggests that this variable behaves as a mediator and not as a moderator.

### Hypothesis E: fear, anxiety, and post-traumatic stress are related to depression (see Fig. 1e)

This model assumes the same assumptions as to the previous hypothesis (model D) but considers that fear of COVID-19 and depression are directly related. It should be noted that although fear of COVID-19 and depression in bivariate analyses have found a positive but low correlation [5], this correlation may increase if mediated by factors such as anxiety or post-traumatic stress. Additionally, by considering all possible relationships, it is possible to see more clearly which dimensions are more or less related.

A better understanding of the relationship of the triad of fear, anxiety, and post-traumatic stress with the appearance of depressive symptoms will allow the identification of how these variables would trigger emotional problems during the context of the COVID-19 pandemic. An important element to consider is that the model could be affected if the groups evaluated have a higher exposure to or knowledge about the virus. Therefore, it is necessary to identify whether the models evaluated are equivalent among health-care workers and the general population. Because the former has greater knowledge of the treatment and evolution of the virus and greater average exposure to the virus due to the nature of their work, the relationships between the variables of the triad with depressive symptoms could be affected.

Therefore, there are two objectives related to the previously presented hypotheses. The first is to determine if “hypothesis A” about the relationship between the fear of COVID-19, anxiety, and post-traumatic stress fits adequately with the data collected from the general population and health-care workers (see Fig. 1a). The second is to determine which of the hypotheses presented above best explains the relationship between depression and the triad of fear, anxiety, and post-traumatic stress in both groups (see Fig. 1b-e). Our study considers a differentiated assessment of the general population and healthcare workers as they are exposed to different concrete and unspecific conditions of fear, anxiety, and stress.

## Methods

### Study design

A cross-sectional study was conducted that included the use of self-reported questionnaires. An online survey was used to avoid physical contact and the spread of SARS-CoV-2 among participants. Demographic and social data of the participants were obtained. Anxiety, fear of COVID-19, depression, and post-traumatic stress were measured using validated questionnaires and scoring systems. Data collection took place over 1 week, April 17–23, 2020, 1 month after the state of emergency was declared and mandatory self-quarantine was ordered in Peru. The sampling was not probabilistic and we used networks of contacts through social networks and other digital media to circulate the online survey.

### Participants

Information was collected from adults living in Lima, the capital of Peru and the most populous city in the country. Inclusion criteria included: 18 to 80 years of age and an agreement to participate in the online survey. The participants were divided into two groups: the general population and health-care workers. Data collection sought to provide a sufficient number of cases from each group to perform the analyses (at least 150 participants per group) [21].

### Variables and measurement instruments

#### Fear of COVID-19

The Fear of COVID-19 Scale (FCV-19S) is a one-dimensional scale with seven items used to assess fears of COVID-19 in the general population. The items are scored on a 5-point scale ranging from 1 (strongly disagree) to 5 (strongly agree). Total scores range from 7 to 35, indicating that, with higher scores, fear of COVID-19 is increased [22]. The reliability values of the scores for internal consistency were acceptable to α = 0.82. The evaluation properties of the instrument have been evaluated in a previous study [5]. There is evidence of validity and reliability in their scores.

#### Symptoms of post-traumatic stress

The Impact of Event Scale-Revised (IES-R) was used, with 22 items scored with a five-point scale, ranging from 0 (none) to 4 (extremely) [23]. The IES-R is a self-report scale of three dimensions: a) intrusion dimension which evaluates indicators of intrusive thoughts, nightmares, intrusive feelings and images, and a new dissociative-type experience (item 1, 2, 3, 6, 9, 14, 16, and 20); b) avoidance dimension which is used to evaluate indicators of numbness and avoidance of feelings, situations, and ideas (item 5, 7, 8 11, 12, 13, 17, and 22); c) hyperarousal dimension which analyzes indicators of anger, irritability, hypervigilance, difficulty concentrating, and intensified startle response (item 4, 10, 15, 18, 19, and 21). This instrument has shown good internal consistency (α = 0.964). The three dimensions are summed and present an overall score with a cohort point of 33 or more points corresponding to post-traumatic stress symptoms [24]. The premise used to define the highly stressful IES-R event was “respond based on your experience of the COVID-19 pandemic.

#### Depressive symptoms

The Patient Health Questionnaire-9 (PHQ-9) was used to evaluate depressive symptoms. The PHQ-9 is a self-administered scale, scored from 0 (nothing) to 3 (almost every day), that consists of nine items based on the nine indicators of major depression from the DSM-IV [25]. Their scores range from 0 to 27, with a cohort point of 10 being considered as the presence of clinically relevant depressive symptoms [26]. The validation of the PHQ-9 conducted in Peru has shown adequate levels of reliability and validity for a single-dimensional model of the PHQ-9 [27].

#### Anxious symptoms

The Generalized Anxiety Disorder Scale-7 (GAD-7) is a valid and effective self-reporting instrument for assessing the severity of anxiety disorders in clinical practice [28]. A cohort point of 10 or more is considered to correspond to clinically relevant anxious symptoms [29]. The scale has been previously translated into Spanish and validated [30]. It consists of seven items designed to measure the symptomatology of anxiety during the 2 weeks before self-application. Each item is scored on a Likert scale ranging from 0 (nothing) to 3 (almost every day). In the present study, the GAD-7 had adequate internal consistency (Cronbach’s alpha = 0.898). Generalized anxiety was assessed, rather than anxiety related to COVID-19, since it corresponds to a global anxiety response (i.e., job loss, illness, family problems) and not only related to the virus infection.

#### Socio-demographic characteristics

Socio-demographic information was collected on the following: sex (man or woman), civil status (married, divorced, single, or widowed), educational level (primary, secondary, technical, or university), employment status (formal employment, informal employment, or unemployed), if they profess a religion (yes/no), and if they self-report having a mental health problem (yes/no). Age was recorded as a continuous variable and categorized into six groups of 10 years each (18–19, 20–29, 30–39, 40–49, 50–59, and 60 or more). Additionally, the type and number of COVID-19 symptoms were considered for the creation of a variable based on whether the person reported having a cough, tiredness, muscle pain, headache, or diarrhea. This data was considered within the collection of information concerning the fact that previous research has considered the presence and severity of covid symptoms as a characteristic that can favor the appearance of reaction levels of emotional distress, stress, anxiety, depression, and PTSD [31, 32] as populations with worse health conditions may experience greater psychological vulnerability due to uncertainty about their health status, follow-up, treatment and care [3].

### Procedures

Data collection focused on collecting a sufficient number of participants for the general population and health professionals, so the questionnaire was socialized through social networking groups specific to health workers and through profile pages where there is a greater reach to the general population.

### Data analysis

#### Descriptive and prevalence

A descriptive analysis was conducted for the general population and health-care workers. Also, the prevalence of clinically relevant depressive symptoms (PHQ-9 ≥ 10 points) [26], clinically relevant anxious symptoms (GAD-7 ≥ 10 points) [29], and post-traumatic stress symptoms (IES-R ≥ 33 points) [24] was analyzed.

#### Relation between variables and reliability

The correlation between variables was determined by Spearman (*r*_*s*_) since the normality assumptions were not fulfilled. A large (*r*_*s*_ > 0.70), moderate (*r*_*s*_ > 0.50), or small (*r*_*s*_ > 0.30) ratio is determined based on the size of the correlation coefficient. In addition, reliability was evaluated with the omega coefficient, considering adequate values greater than 0.80 [33].

#### Structural equation model

A structural equation model was used using the weighted least squares means and variance adjusted (WLSMV), which allows handling non-normality [34]. This analysis used the polychoric matrices suitable for the ordinal nature of the items [35, 36]. All of the models presented in Fig. 1 were evaluated. First, we evaluated a baseline model that supports the relationship between fear of COVID-19, anxiety, and post-traumatic stress (model A) to have evidence that it is a model with adequate adjustment based on which to evaluate the more complex models. This model was taken as a baseline because it presents sufficient evidence to support it, as described in the background section. Then models 1B through 1E were evaluated to see which model had the best fit and most variance.

It should be noted that the model was adjusted using two socio-demographic variables (sex, age, and symptoms of COVID-19). First, sex was added to the model to influence fear of COVID-19 since the fear of the COVID-19 scale and is not invariant between men and women [5]. Thus, the sex and age variables were added so that the model could be adjusted. Second, anxiety symptoms were adjusted by the COVID-19 symptoms because there are other instruments focused on measuring anxiety-related to COVID-19 that have demonstrated that the anxiety experienced is directly related to the perception of COVID-19 symptoms (i.e., cough, dizziness, muscle pain) [37]. Therefore, it was considered necessary to include these three socio-demographic variables in the model analysis.

Two criteria were used to evaluate the different models. First, different goodness-of-fit indices were evaluated. We used the Comparative Fit Index (CFI) and the Tucker-Lewis Index (TLI), both with appropriate values ≥0.90. The Standardized Root Mean Square Residual (SRMR) and the Root Mean Square Error of Approximation (RMSEA) with a confidence interval of 90% and with adequate values < 0.08 were used to compare model fit [34, 38]. Second, the *R*^*2*^ of the outcome variable (depressive symptoms) was evaluated, which allows us to know how much variance explains the proposed model. The models that explain the most variance are the most adequate [17].

In addition, mediation analysis assessed the indirect effect of posttraumatic stress on the anxiety-depression relationship. The cross-product of the coefficients was calculated to obtain an indirect effect of the structural model with 5000 iterations were used to calculate the standard error and obtain the path of the indirect effect.

### Statistic software

All analyses were done in R Studio, with the packages “lavaan,” “semTools,” and “semPlot.”

### Ethics

The study protocol and the instruments used for the evaluation were approved by the ethics committee of the Universidad San Martin de Porres (Oficio No. 227–2020-CIEI-FMH-USMP).

## Results

### General characteristics and prevalence

The characteristics of the participants are found in Table 1. The average age of health workers was 38.8 (SD = 11.2) and of the general population was 38.3 (SD = 13.2). The majority of participants were women. The overall prevalence for health-care workers and the general population was 16% for clinically relevant depressive symptoms, 11.7% for clinically relevant anxious symptoms, and 14.9% for post-traumatic stress. It was found that 23.1% of the participants had one of these mental health problems. A higher prevalence of depressive, anxious, or stress symptoms was identified in the general population (28.6%) compared to health-care workers (17.9%).

**Table 1:** Socio-demographic characteristics (*n* = 830)

		General population (*n* = 640)		Health-care workers (*n* = 190)	
		n	%	n	%
Age	18 to 19	20	3.1%	0	0.0%
	20 to 29	180	28.1%	38	20.0%
	30 to 39	190	29.7%	85	44.7%
	40 to 49	103	16.1%	32	16.8%
	50 to 59	94	14.7%	17	8.9%
	60 to more	53	8.3%	18	9.5%
Sex	Men	217	33.9%	68	35.8%
	Women	423	66.1%	122	64.2%
Civil Status	Married	260	40.6%	85	44.7%
	Divorced	56	8.8%	15	7.9%
	Single	317	49.5%	89	46.8%
	Widowed	7	1.1%	1	0.5%
Educational level	Primary	1	0.2%	0	0.0%
	Secondary	81	12.7%	0	0.0%
	Technical	109	17.0%	3	1.6%
	University	449	70.2%	187	98.4%
Employment status	Formal employment	381	59.5%	176	92.6%
	Informal employment	88	13.8%	8	4.2%
	Unemployed	171	26.7%	6	3.2%
Do you have a religion?	No	211	33.0%	47	24.7%
	Yes	429	67.0%	143	75.3%
Diagnosis of a mental health problem	No	553	86.4%	162	85.3%
	Yes	87	13.6%	28	14.7%
Number of inseparable symptoms of COVID-19^a^	None	435	68.0%	132	69.5%
	1	135	21.1%	40	21.1%
	2	40	6.3%	14	7.4%
	3 to more	30	4.7%	4	2.1%
Depression	No	514	80.3%	167	87.9%
	Yes	126	19.7%	23	12.1%
Anxiety	No	547	85.5%	174	91.6%
	Yes	93	14.5%	16	8.4%
Stress	No	514	80.3%	170	89.5%
	Yes	126	19.7%	20	10.5%

^a^Cough, fatigue, muscle pain, headache, or diarrhea

### Relationship between variables and reliability

The relationship between depressive and anxious symptoms was high and very similar in the general population and health-care workers (*r*_*s*_ > 0.70). The general population presented a strong relationship between anxious symptoms and post-traumatic stress (overall score and three dimensions), while health-care workers reached a moderate relationship (*r*_*s*_ > 0.50) [39]. However, these values did not change much (see Table 2). All the instruments evaluated presented adequate levels of internal consistency.

**Table 2:** Relationship between the fear of COVID-19, depressive symptoms, anxious symptoms, and post-traumatic stress (*n* = 830)

		(1)	(2)	(3)	(4)	(4.1)	(4.2)	(4.3)	ω
General population (*n* = 640)	(1) Fear of COVID-19	1							0.90
	(2) Depressive symptoms	0.32	1						0.88
	(3) Anxious symptoms	0.44	0.73	1					0.89
	(4) Post-traumatic Stress	0.54	0.65	0.73	1				0.98^a^
	(4.1) Intrusion	0.55	0.63	0.71	0.94	1			0.82
	(4.2) Avoidance	0.51	0.56	0.64	0.95	0.83	1		0.93
	(4.3) Hyperarousal	0.49	0.67	0.74	0.92	0.86	0.80	1	0.91
Health-care workers (*n* = 190)	(1) Fear of COVID-19	1							0.91
	(2) Depressive symptoms	0.35	1						0.87
	(3) Anxious symptoms	0.48	0.74	1					0.90
	(4) Post-traumatic Stress	0.61	0.56	0.64	1				0.98^a^
	(4.1) Intrusion	0.59	0.54	0.62	0.93	1			0.79
	(4.2) Avoidance	0.57	0.46	0.55	0.94	0.80	1		0.93
	(4.3) Hyperarousal	0.54	0.64	0.69	0.91	0.84	0.77	1	0.87

The relationship was evaluated with Spearman’s coefficient. All values are significant (*p* < 0.001). ^a^ Omega coefficient considering second-order models

A moderate relationship (*r*_*s*_ > 0.50) was found between post-traumatic stress (overall score and three dimensions) with fear of COVID-19 and depressive symptoms for both the general population and health workers. It should be noted that a small relationship was found between fear of COVID-19 and depressive and anxious symptoms in both groups (*r*_*s*_ > 0.30).

### Structural equation model

The “model A” or baseline model of the relationship of fear of COVID-19, anxiety, and post-traumatic stress showed adequate goodness-of-fit indices (see Table 3), indicating that this model is stable and could be used to evaluate more complex models to explain depressive symptoms in health-care workers and the general population. Therefore, models to explain depressive symptoms were evaluated using “model A” as a basis (see Fig. 1b-e).

**Table 3:** Goodness-of-fit indices of the structural equation model (*n* = 830)

		*X*^2^	*df*	CFI	TLI	RMSEA [90% CI]	SRMR	*R*^*2*^
Both groups (*n* = 830)	Model A^a^	3389.7	692	0.940	0.945	0.068 [0.066–0.071]	0.056	–
	Model B	4821.9	1069	0.923	0.928	0.065 [0.063–0.067]	0.065	0.613
	Model C	4741.9	1068	0.924	0.930	0.064 [0.062–0.066]	0.063	0.682
	Model D^a^	4120.2	1068	0.937	0.942	0.059 [0.057–0.061]	0.056	0.739
	Model E	4114.4	1067	0.937	0.942	0.059 [0.057–0.061]	0.055	0.748
General population (*n* = 640)	Model A	2674.9	692	0.946	0.951	0.067 [0.064–0.070]	0.058	–
	Model B	3716.9	1069	0.933	0.938	0.062 [0.060–0.064]	0.065	0.627
	Model C	3672.3	1068	0.934	0.939	0.062 [0.060–0.064]	0.064	0.689
	Model D	3262.0	1068	0.945	0.949	0.057 [0.054–0.059]	0.057	0.750
	Model E	3262.5	1067	0.945	0.949	0.057 [0.055–0.059]	0.057	0.759
Health-care workers (*n* = 190)	Model A	1196.5	692	0.956	0.960	0.062 [0.056–0.068]	0.070	–
	Model B	1859.8	1069	0.936	0.941	0.062 [0.057–0.067]	0.089	0.555
	Model C	1807.4	1068	0.940	0.945	0.060 [0.055–0.065]	0.086	0.705
	Model D	1664.6	1068	0.952	0.955	0.054 [0.049–0.059]	0.078	0.716
	Model E	1662.7	1067	0.952	0.955	0.054 [0.049–0.059]	0.078	0.723

**Fig. 2:**
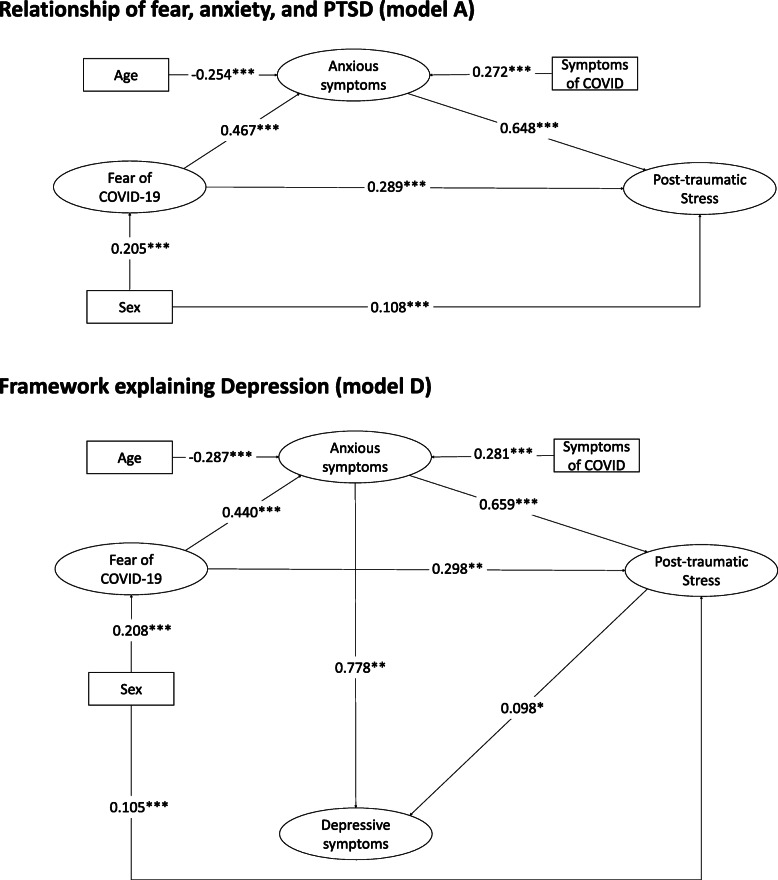
Model of the relationship of fear of COVID-19, anxiety, and post-traumatic stress (model A) and the framework explaining depression (model D) for all participants (*n* = 830). Note: *p < 0.05,**p < 0.01, ***p < 0.001

*X*^*2*^ chi-square, *df* degree of freedom, *CFI* comparative fit index, *TLI* Tucker-Lewis Index, *RMSEA* root mean square error of approximation, *SRMR* standardized root mean square, *R*^*2*^ coefficient of determination. ^a^These are the models presented in Fig. 2

It was identified that the four models evaluated (Model B to E) identified adequate goodness-of-fit indices in all cases. However, the models that most explained the depressive symptoms were model D (explaining 73.9% of the variance) and model E (explaining 74.8% of the variance). Although model E contributed 0.7% more variance, the relationship between fear of COVID-19 and depressive symptoms was − 0.104. This finding would be contrary to what is theoretically expected since it is estimated that the greater the fear of the COVID-19, the greater the levels of depressive symptoms are expected, as was found in the bivariate analysis, where these variables presented a direct, significant, and positive correlation (see Table 2). Therefore, it was decided that model D would best explain the depressive symptoms because it presents sufficient goodness-of-fit indices, explains almost the same amount of variance as model E, and fits best with the theoretical assumptions (see Fig. 2).

**Fig. 3:**
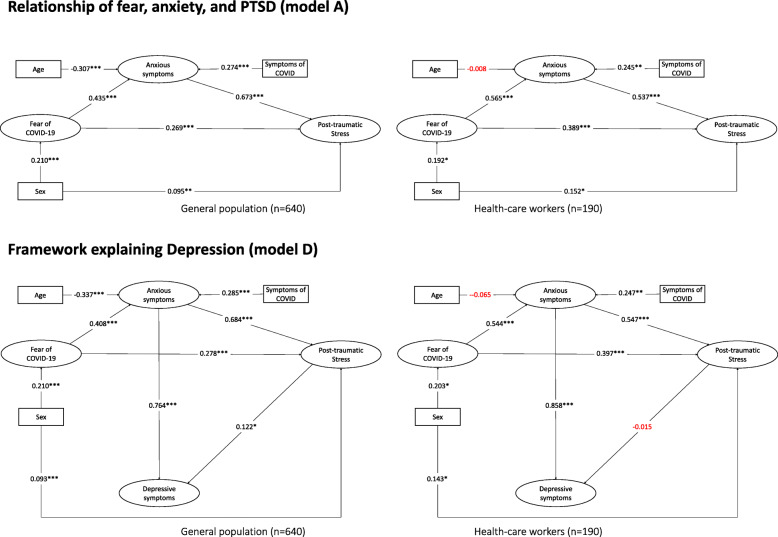
Model of the relationship of fear of COVID-19, anxiety, and post-traumatic stress (model A) and the framework explaining depression (model D) for the general population and health-care workers. Note: *p < 0.05,**p < 0.01, ***p < 0.001. Values in red are not significant

Analysis of model D for the total number of participants, the general population, and healthcare workers (see Fig. 2 and Fig. 3), identified that fear of COVID-19 is directly associated with anxiety (*p* < 0.001), and with the post-traumatic stress response (*p* < 0.001). Also, anxiety is directly associated with post-traumatic stress and depressive symptoms (*p* < 0.001). When the relationships between the variables in model D were evaluated by type population, it was found that the general population and health professionals had very similar values between the variables (see Fig. 3). However, some variables are no longer significant between the two groups. On the one hand, age in health professionals does not associate with anxiety levels (β = − 0.065; *p* = 0.479), while in the general population it does (β = − 0.337; p < 0.001). On the other hand, post-traumatic stress is not associated with the presence of depressive symptoms in the general population (β = 0.122; *p* = 0.025), but not in healthcare professionals where it presents a very small and non-significant coefficient (β = − 0.015; *p* = 0.876).

Mediation analysis identified that in the overall participants the indirect effect of post-traumatic stress on the relationship of anxiety and depression was significant (β = 0.10; 95%CI = 0.05 to 0.16). However, the results varied depending on the population assessed. In the general population, post-traumatic stress mediated the relationship between anxiety and depression (β = 0.12; 95%CI = 0.06 to 0.18), but the mediation was not significant in health care workers (β = 0.03; 95%CI = − 0.11 to 0.19).

A sub-analysis was conducted to assess the role of other sociodemographic variables such as sex and age with COVID-19 fear, anxiety, and post-traumatic stress variables. It was identified that only significant relationships were between fear of COVID-19 and age; post-traumatic stress and sex; and age and anxious symptoms.

## Discussion

### Main findings and significance of the results

Our study proposes and tests different models based on structural equations models that allowed testing hypothetical models based on substantive theories that use the triad of fear, anxiety, and post-traumatic stress to explain depressive symptoms in the general population and health-care workers during the COVID-19 pandemic.

We found that this triad, along with three additional variables (number of COVID-19 symptoms, age, and sex), explained more than 71% of depressive symptoms in the general population and health care workers. The proposed model (model D) allows us to understand the role of fear of COVID-19, age, and the number of perceived COVID-19 symptoms in the presence of anxiety symptoms. In turn, it is found that anxiety symptoms and sex affect the stress symptoms, and all of these are associated with depressive symptoms. Anxiety symptoms are the most influential in the occurrence of depressive symptoms, in compassion to stress. It was identified that the model presents a different behavior for the general population and health-care workers since in the latter group the variables of age and post-traumatic stress are not associated with depressive symptoms.

This explanatory model can be used in health contexts and populations for how emotional factors (fear, anxiety, and stress) can affect depressive symptoms. Also, the model can be used to understand a differential response of health-care workers and the general population in the genesis of depressive symptoms.

Analysis of the triad of fear-anxiety-stress to predict depressive symptoms identified that only anxiety is strongly associated with depressive symptoms, as PTSD has a small effect, and there is no direct relationship with fear with COVID-19. However, our study was supported by the fact that this triad mechanism represents a joint response to emotionally charged events such as a pandemic. Therefore, we consider that its assessment as a whole (fear, anxiety, and stress) may better explain the presence of depressive symptoms.

Also, there is a higher prevalence of depressive, anxious, and stress symptoms in the general population than in health workers. Therefore, it is necessary to focus on mental health interventions and prevention activities for both groups.

### Contrasting findings with existing literature

#### Prevalence

In a previous study to compare the emotional effects of COVID-19 among three different groups in Peru was found a prevalence of clinically relevant symptoms of depression in general population (21%), healthcare workers (9%), and healthcare workers in COVID-19 areas (8%) [40]. The prevalence recorded in the systematic review studies was higher than the values recorded in our research for depression (33.7 to 22.8%), anxiety (29.6 to 23.2%), and post-traumatic stress (31.9%) [3, 41, 42]. However, it should be considered that the methods used to assess prevalence in systematic reviews are heterogeneous, belong to different times of the pandemic, and mostly correspond to high-income countries. Therefore, these elements could be overestimating the values.

The prevalence of each variable reported in our study is higher than those recorded in national studies conducted in Peru before the pandemic, where the prevalence of depression was 6.4% [43]. Although the pre-pandemic prevalence of depression in Peru is estimated to have been stable and not increasing [44], the national prevalence has likely increased during the pandemic. The increase in the prevalence is especially true for people who are aware of having a chronic disease [45], as they are at-risk populations.

#### Structural equation model and relationship between variables

Only two studies have been identified that pose predictive models that include fear of COVID-19, anxiety, stress, and depressive symptoms. This gap is understandable due to the limited number of published and pre-print studies as the pandemic, along with its related factors, is an emerging issue.

The first study was carried out on Ecuadorian university students and proposed a model that predicts the depressive symptoms, from the fear of the COVID-19, anxiety, and stress [46]. This study agrees with our findings, and although the model presented is not the same as ours, it reinforces the hypothesis that the triad of fear, anxiety and stress predicts depressive symptoms. However, this study has two significant limitations. First, the instrument used to measure the main outcome is the Depression Anxiety Stress Scales (DASS), which presents good performance when used as a bifactorial instrument (a global dimension), but its performance presents inconsistencies when used as a three-dimensional correlated instrument (original DASS model) [47]. This inconsistency could introduce biases in the measurement of the main outcome. Second, the study does not evaluate other possible predictive models that could have a better fit. Furthermore, it is not clear what the process to define the model presented was. For example, it is not justified because the fear of COVID-19 and stress are not related, and studies have identified a strong relationship between both variables [48, 49].

The other study carried out in pregnant women evaluates a model where fear and anxiety related to COVID-19 predict the appearance of mental health problems, which were evaluated with the DASS but considering only an overall score that adds up the scores of anxiety, depression, and stress [50]. Although not directly comparable, this study identifies that fear of COVID-19 plays an essential role in the presence of mental health problems.

Some studies have been identified that partially analyze our proposed model. One study finds that fear of COVID-19 has an indirect effect on the presence of depressive symptoms [51], and another study in general population identified that fear of COVID-19 has an association with mental health problems (i.e., anxiety and depression) [52], which supports our conclusions. Also, another study has reported a strong relationship between anxiety, stress, and depression in the context of the COVID-19 pandemic [49].

Other studies have evaluated variables that were not included in the study but may have a relevant role in the triad of fear, anxiety, and post-traumatic stress with depression. Two studies have evaluated the mediated role that uncertainty tolerance may play with COVID-19 fear and depressive symptoms [53, 54]. Although this may be a limitation, our study evaluates in a more complex way the role that anxiety and stress have, unlike the mentioned studies. Furthermore, the mediating role of anxiety within the relationship between fear of COVID-19 and depression is not considered in the mentioned studies.

The proposed model shows a different behavior between health professionals and the general population. On the one hand, different studies have identified that age is negatively associated with anxious symptoms in the general population and is more frequent in younger people [55, 56] and that the general population has higher levels of anxiety and depression than health professionals [57]. A possible explanation for the decrease in the strength of the relationship between age and anxiety symptoms is that health care workers are exposed to less uncertainty about the virus (because of their biomedical training) and that they are better able to dissipate false news about COVID-19, which occurred at the beginning of the pandemic. On the other hand, the association between post-traumatic stress and depressive symptoms was not significant in health care workers but was significant in the general population. We have identified two possible explanations. First, although health care workers have had greater exposure to the virus on average, their biomedical training could be a factor that helps them to reduce their uncertainty about the virus and therefore have less stress [53]. Second, the fact of working itself is a factor that is associated with a greater perception of self-efficacy, and this is a protective factor for mental health problems such as stress or depression [52]. Both elements could be reducing the strength of the association between post-traumatic stress and the presence of depressive symptoms. In contrast, the general population, because they are on average not working, do not have a basic biomedical education, may experience greater uncertainty, have difficulty differentiating false news related to the virus, and thus experience more post-traumatic stress and depressive symptoms.

### Implications in public health and making decisions

Previous studies show the key role that organizations and public health bodies play in promoting adaptive coping and reducing health worries and the emotional and psychological distress caused by the pandemic. Evidence highlights particular groups at risk of developing mental health problems (contact with infected patients, having children), and time points where risk may increase (initial response phase, when quarantined) [58]. Our study raises three main implications for public and global health in Peru and other Low and Middle-Income Countries (LMIC) with similar characteristics. First, the high prevalence of mental health problems recorded during the pandemic [3] makes it necessary to establish national policies and strategies for screening and epidemiological surveillance of the components of the triad of fear of COVID-19, anxiety, and post-traumatic stress, since these three elements predict the presence of depressive symptoms and other emotional problems. Health-care workers from Peru and other LMICs are overburdened by the pandemic and are in a fragmented health system [59]. It is recommended that technological tools such as apps or short (2 or 4 items) virtual self-reporting systems be used to collect information on emotional problems (i.e., anxiety, stress, fear, or depression) from the general population and health workers. These strategies have proven useful for addressing mental health needs and referring users with severe emotional problems in other countries during a pandemic emergency. Second, it is recommended to develop and implement preventive activities focused on the three elements of the triad (fear of COVID-19, anxiety, and post-traumatic stress). It is not only necessary to provide care to people who have moderate or severe mental health problems (depression, anxiety, or post-traumatic stress) but also to develop strategies aimed at people with mild and non-specific mental health indicators such as stress or fear of COVID-19 [60]. These strategies could prevent the latter individuals from evolving to a more advanced stage involving significant health problems and generating years of life lost due to disability [60]. Third, in Peru and other LMICs, the treatment gap for mental health problems such as depression is high, and it is estimated that only 1 in 10 people who require treatment receive it [44]. This gap may have increased during the pandemic, so actions to increase access to appropriate care need to be targeted. This model can be used to identify cases with a high fear of COVID-19 and facilitate their access to the health care system so that cases with greater severity of depression do not develop in the future.

At the level of mental health decision-makers, increased resources in mental health care are strongly recommended. In Peru, only 2% of all GDP is allocated to health, and only 0.2% of GDP is allocated to mental health [61], thus requiring increased human, financial, and political capital resources to improve mental health during and after the COVID-19 pandemic.

### Strengths and limitations

Our study presents and assesses different models for understanding depressive symptoms using the triad of fear, anxiety, and stress. Our model applies a solid framework on the influence of this triad on mental health and applies it in the presence of depressive symptoms [6–9]. Another strong point of the study is the use of statistical methods that consider all these variables within their analysis. However, our study is not free of limitations. First, this cross-sectional study was conducted during the beginning of the first wave of COVID-19 in Peru (April 2020), so this model could vary during the later stages of the pandemic. Second, the data are from an urban city with the highest population density in the country (Lima, the capital of Peru), so the results could vary in a rural or less densely populated context (risk of infection). Third, the prevalence of depressive, anxious, and stress symptoms was evaluated with validated psychometric instruments, but this evaluation is not a substitute for clinical assessment, so misclassification can be a problem. Fourth, the scale for measuring PTSD (IES-R) is based on the criteria of the DMS-IV, which proposes three dimensions (intrusion, avoidance, and hyperactivity). However, the criteria for defining PTSD is modified in the DSM-5 where four dimensions are proposed (Reexperiencing, Avoidance, Negative Alterations in Cognitions and Mood, Hyperarousal), therefore, it is possible that the PTSD construct is being partially measured. This is not the case in the instruments used to measure depression and anxiety since the DMS-IV and DSM-5 criteria are equivalent. Fifth, other variables that could be useful to explain the model such as intolerance of uncertainty [53], pregnancy in the female participants [50], anxiety caused by COVID-19 [50], or income level [16] could not be included. Sixth, our study is a cross-sectional study so that causality statements cannot be made only about the association. Seventh, no differentiation was made between front-line staff working directly with COVID-19 patients and other types of health professionals who may be doing remote work. Thus, it is possible that not all health care workers had the same level of exposure to the virus.

## Conclusions and recommendations

Our study concludes that the triad of fear, anxiety and post-traumatic stress may explain more than 70% of depressive symptoms in the general population and health care workers during the COVID-19 pandemic. Also, a higher prevalence of depressive, anxious, and stressful symptoms is identified in the general population than in health care workers.

Researchers and decision-makers are encouraged to develop and implement policies and strategies to conduct screening for and epidemiological surveillance of fear of COVID-19, anxiety symptoms, and stress, as these variables, are predecessors to depressive symptoms. Another recommendation is the development and implementation of preventive actions for these three elements (fear, anxiety, and stress) in the general population and health-care workers so that the prevalence of depressive symptoms can be reduced.

## Data Availability

The database is available from 10.6084/m9.figshare.13683955
